# Simultaneous onset of systemic sclerosis and light chain amyloidosis: the first report of a case report and review of the literature

**DOI:** 10.1007/s00296-025-05982-5

**Published:** 2025-09-16

**Authors:** Anna Colangelo, Francesco Tromby, Elisabetta Agliani, Lorenza Bruno, Giacomo Cafaro, Federico Crusco, Anna Mengoni, Cinzia Zuchi, Roberto Gerli, Elena Bartoloni, Carlo Perricone

**Affiliations:** 1 Section of Rheumatology, Department of Medicine and Surgery, University of Perugia, Piazzale Giorgio Menghini, 1, Perugia, PG 06129 Italy; 2 Cardiology and Cardiovascular Pathophysiology, S. Maria della Misericordia Hospital, University of Perugia, Perugia, Italy; 3 USL Umbria 2 “Nuovo Ospedale San Giovanni Battista”, Radiologia, Foligno, Italy; 4 Hematology and Bone Marrow Transplantation Unit, Department of Medicine and Surgery, University of Parma, Parma, Italy

**Keywords:** Systemic sclerosis, Amyloidosis, Light-chain immunoglobulin, anti-CD38, Daratumumab

## Abstract

Systemic sclerosis (SSc) and amyloidosis are rare, complex conditions that impair the function of multiple organs, each with distinct pathogenic mechanisms: autoimmunity for SSc and misfolded protein deposition for amyloidosis. We present the first documented case of a 57-year-old woman with coexisting SSc and systemic AL amyloidosis with multi-organ involvement, in which treatment for amyloidosis led to a notable improvement in SSc symptoms. The patient presented experiencing fatigue, exertional dyspnea, epigastric pain and syncopal episodes in the summer of 2023. Investigations revealed mild increase in left ventricle thickness, elevated NT-proBNP and troponin with negative coronary angiography. She was subsequently diagnosed with SSc with multi-organ involvement and systemic AL amyloidosis confirmed by biopsy. Treatment with a modified Dara-CyBorD protocol led to improvement in SSc symptoms, especially in terms of dyspnea and skin involvement. This is the first reported case of SSc coexisting with systemic AL amyloidosis. The patient responded well to therapy for amyloidosis, suggesting potential overlapping treatment benefits. A multidisciplinary approach was essential, and further studies are needed to explore therapeutic interactions between these two rare diseases.

## Introduction

Systemic sclerosis (SSc) is a complex autoimmune disease characterized by cutaneous and internal organ fibrosis, diffuse fibroproliferative vascular modifications, and autoimmunity. The pathogenesis involves an initial vascular injury due to autoimmunity and environmental factors, leading to chronic inflammation and fibroblast activation, culminating in fibrosis across multiple organs [1, 2].

AL (light-chain) amyloidosis is a systemic disorder caused by the extracellular deposition of misfolded immunoglobulin light chains produced by a clonal population of plasma cells. These amyloid fibrils infiltrate multiple organs, most commonly the kidneys, heart, liver, and peripheral nerves, leading to progressive organ dysfunction. The clinical presentation of AL amyloidosis is notoriously variable and often overlaps with features of other chronic conditions, making timely diagnosis particularly challenging [3].

The coexistence of SSc and systemic AL amyloidosis is exceptionally rare and poses significant diagnostic and therapeutic challenges. Both diseases can manifest with similar symptoms, such as skin thickening, renal impairment, and cardiac involvement, complicating clinical assessment [4]. Understanding the interplay between these two conditions is critical for appropriate diagnostic evaluation and tailored treatment strategies, particularly given the vastly different pathophysiological mechanisms and therapeutic approaches involved.​.

## Case report

We report the clinical case of a 57-year-old woman who began experiencing fatigue, exertional dyspnea, syncopal episodes and epigastric pain during the summer of 2023. She underwent oesophagogastroduodenoscopy which showed the presence of gastritis and ergometric stress test that was terminated early due to dyspnea and hypotension. Therefore, she was put on the list for coronarography.

**Fig. 1:**
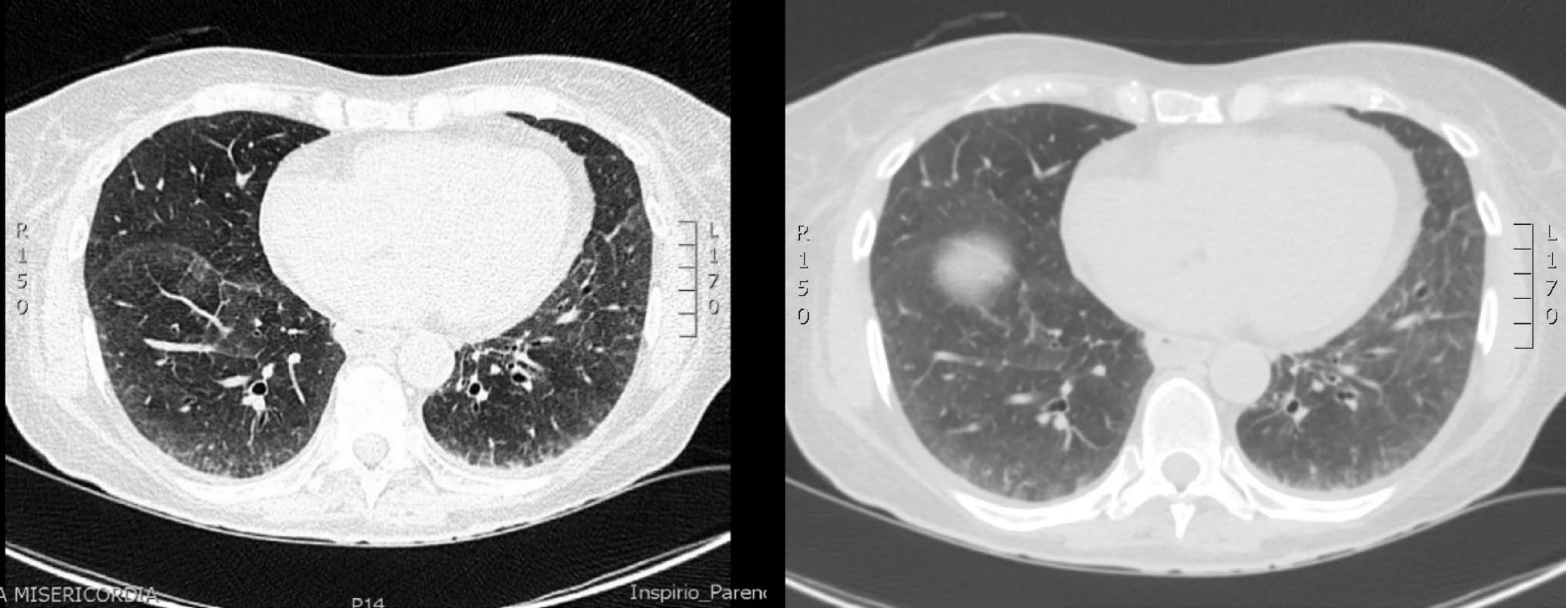
HRCT showing a mosaic attenuation pattern and diffuse ground-glass opacities at the lung bases with interlobular septal thickening

During hospitalisation, the echocardiography showed mild increase in left ventricle (LF) thickness with interventricular septal of 12 mm, not justified by the presence of valvulopathy or systemic arterial hypertension. NT-proBNP was also elevated (1170 pg/ml). Coronary angiography showed no lesions. Serum immunofixation revealed IgM kappa at 6.1 g/L, kappa (κ) light chains at 123 mg/L (normal range: 6–22 mg/L), and lambda (λ) light chains at 53 mg/L (normal range: 5–26 mg/L), with traces of κ light chains on urinary immunofixation. Bone marrow biopsy showed findings consistent with IgM “plasma cell-like” monoclonal gammopathy of undetermined significance (MGUS) without amyloid deposits (Congo red staining and polarized light microscopy were negative). In December 2023, she was admitted again to the cardiology unit due to fatigue, severe dyspnea requiring oxygen supplementation up to 12 L/min, orthostatic hypotension, and elevated troponin levels (1010 ng/L). Echocardiography confirmed mildly increased LV wall thickness with slight of the interventricular septum and preserved EF, with normal filling pressure. High-resolution chest computed tomography (HRCT) showed mosaic attenuation pattern in the middle lobe and lingula, diffuse ground-glass opacities at the bases with interlobular septal thickening and dilated oesophagus (Fig. 1).

Given the onset of rapidly progressive cutaneous involvement characterized by sclerodactyly and thickening of the skin on the distal upper limbs, abdomen, and face with microstomia, rheumatology consultation was requested. Clinical examination revealed a modified Rodnan Skin Score (mRSS) of 11. Laboratory tests were relevant for positive ANA 1:640 with homogeneous staining and anti-topoisomerase I antibodies (anti-Scl70) at high titers (84 U/ml, normal range: <15 U/ml). A capillaroscopy showed an “active” scleroderma pattern. In view of the rapid worsening of the cutaneous manifestations, in addition to a chest HRCT scan, a malignancy workup including an abdominal ultrasound, an esophagogastroduodenoscopy and the test for other autoantibodies such as myositis specific antibodies, anti-RNA Polymerase III, PM/Scl-100, PM/Scl-75, Fibrillarin (U3 RNP) and Th/To antibodies were also performed to rule out paraneoplastic causes and tested negative for underlying malignancy. She was therefore diagnosed with SSc, which also fulfilled ACR/EULAR classification criteria [5] (score 16) with cutaneous, pulmonary, and esophageal involvement. Quite surprisingly, at that time, she had never suffered from Raynaud’s phenomenon (RP) and neither telangiectasia nor digital ulcers were detected at physical examination.

An electromyography (EMG) was also performed, which showed a mild chronic distal sensory-motor polyneuropathy of the lower limbs.

**Fig. 2:**
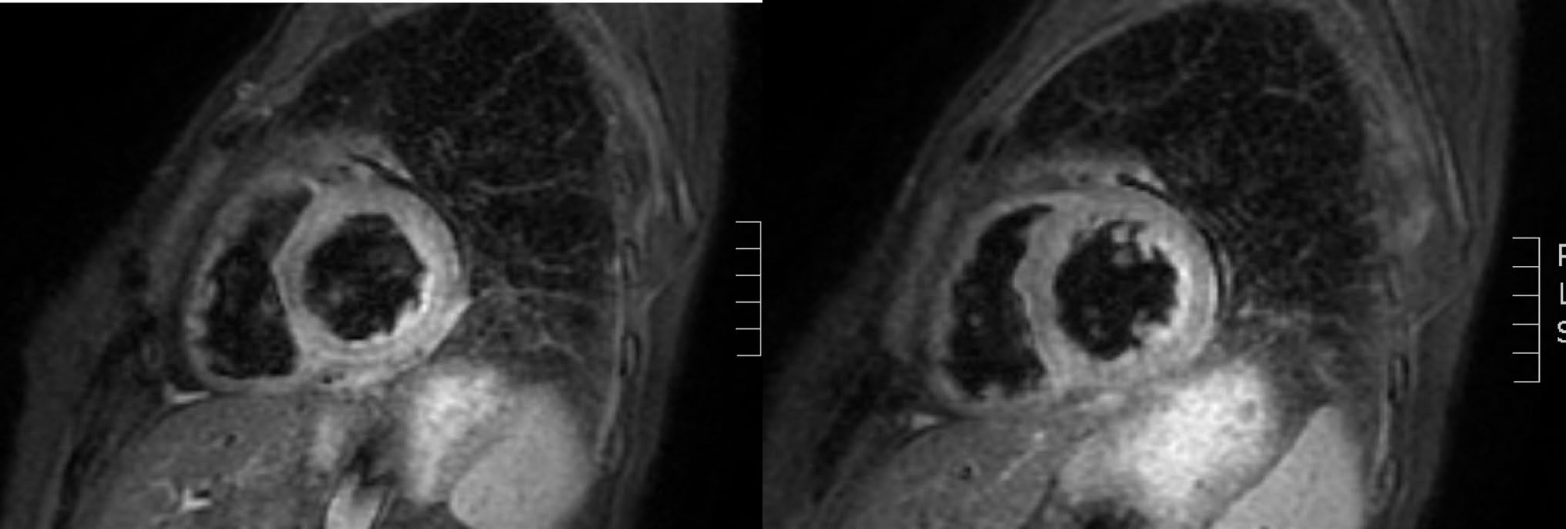
Cardiac MRI findings showing mid-basal left ventricular LGE in a circumferential, subendocardial-to-transmural pattern, more pronounced in the inferior and lateral regions

A periumbilical fat biopsy revealed fibroadipose tissue without amyloid deposits at Congo red and thioflavin staining. Cardiac MRI findings were compatible with amyloidosis showing “mid-basal LV, circumferential, subendocardial-transmural late gadolinium enhancement (LGE), more prominent in the inferior and lateral regions” (Fig. 2).

The MRI pattern, although not pathognomonic, was highly suggestive of cardiac amyloidosis [6]. Interestingly, right ventricular (RV) endomyocardial biopsy revealed histological findings of replacement fibrosis consistent with SSc, while AL amyloid deposits were detected at electron microscopy (EM). EM of the umbilical fat biopsy confirmed AL amyloidosis (κ light chains).

In February 2024, given the poor prognosis of the patient and the concomitant AL amyloidosis, treatment with daratumumab 1800 mg, cyclophosphamide (CYC) 175 mg/m^2^, bortezomib 0.7 mg/m^2^, and dexamethasone 20 mg (Dara-CyBorD protocol) was initiated, using bortezomib at a reduced regimen to minimize cardiac toxicity.

The approach with CYC and its dosage were considered adequate to treat both SSc and AL amyloidosis, requiring cautious monitoring of cardiac parameters. After the first cycle of therapy, troponin levels rapidly decreased to 191 ng/L post-treatment, while NT-proBNP remained stable. Dyspnea significantly improved and O_2_-therapy was reduced to 2 L/min. Autologous haematopoietic stem cell transplantation (ASCT) could not be considered in our patient given the mortality risk of the procedure and the eligibility criteria for stem cell transplant with full dose (200 mg/m^2^) melphalan. Indeed, the criteria for ASCT include, among the others, troponin T < 60 ng/L (higher in our patient), New York Heart Association class < III (III in our patient), systolic blood pressure ≥ 100 mm Hg (lower in our patient).

**Fig. 3:**
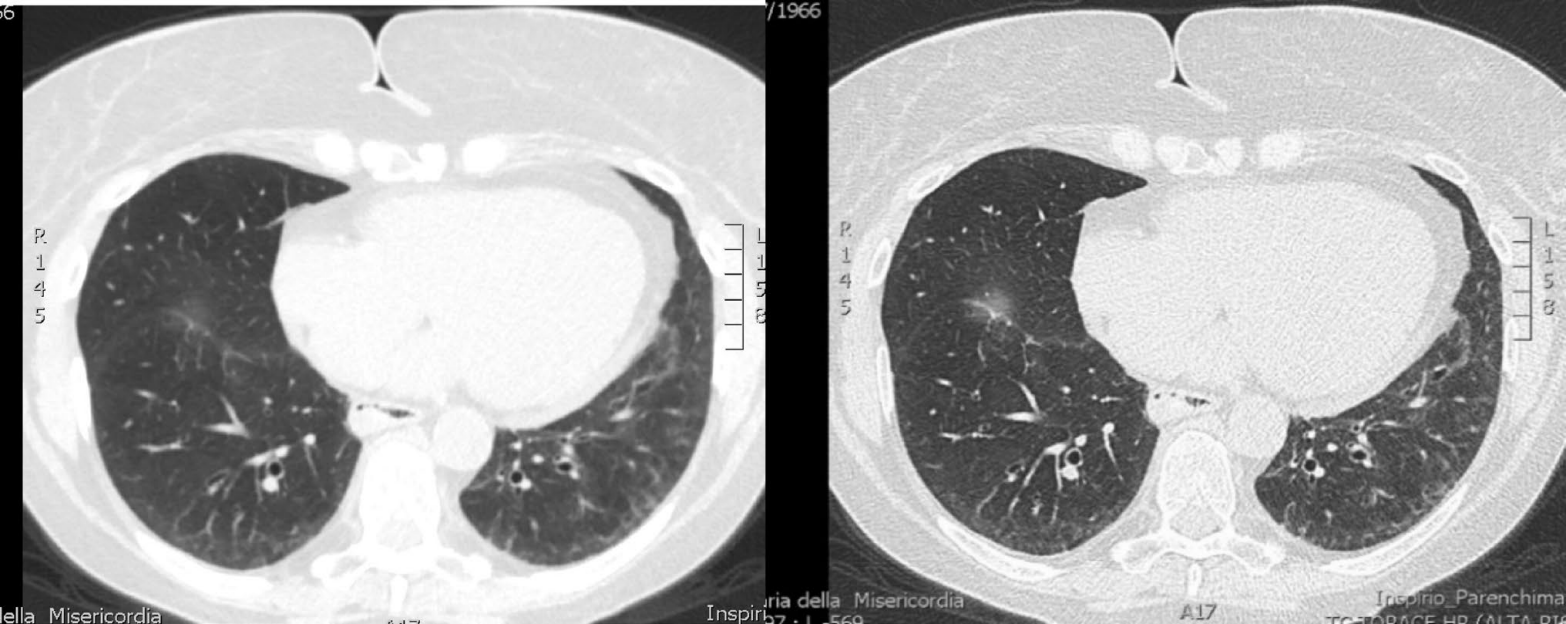
Follow-up HRCT showing a reduction in ground-glass opacities but persistent bilateral basal reticular pattern

After three months of treatment, the patient reported further improvement in dyspnea and fatigue. Follow-up HRCT showed reduced ground-glass opacities but persistent basal reticular patterns bilaterally (Fig. 3).

Repeated cardiac MRI revealed findings indicative of diffuse myocardial involvement by SSc with transmural fibrosis. Right heart catheterization demonstrated moderate post-capillary pulmonary hypertension.

In July 2024, after six cycles of therapy, CYC (total dose approximately 7 g) and bortezomib were discontinued, while daratumumab and dexamethasone were continued. Mycophenolate mofetil (MMF) 2 g/day was therefore initiated along with nintedanib 150 mg twice daily. The latter was at first reduced to 100 mg twice daily and then suspended due to gastrointestinal symptoms (diarrhea) not responsive to antidiarrheal medications and gut modifiers.

In October 2024, a partial improvement in skin manifestations was reported (mRSS = 7) and, remarkably, the patient developed RP. Nonetheless, capillaroscopy remained stable. Control cardiac MRI was performed showing overall stable findings except for a slight improvement in LV ejection fraction and reduction in LV inferolateral wall edema, less noticeable LGE areas in the RV. Further support treatment, including pantoprazole 40 mg/day and nifedipine 20 mg/day, was introduced.

The patient continued to improve, however in January 2025 dexamethasone was discontinued due to osteoporotic vertebral fractures (D12, L1, L2), thus teriparatide was started.

At last visit on August 2025 mRSS was 5, dyspnoea significantly ameliorated, and the HRCT scan performed in June 2025 showed a stable condition. According to Palladini et al. [7], a very good partial haematologic response was obtained for amyloidosis. Since May 2025 daratumumab was also discontinued, and the patient continued maintenance therapy with MMF at a dose of 2 g/day.

### Literature review

**Fig. 4:**
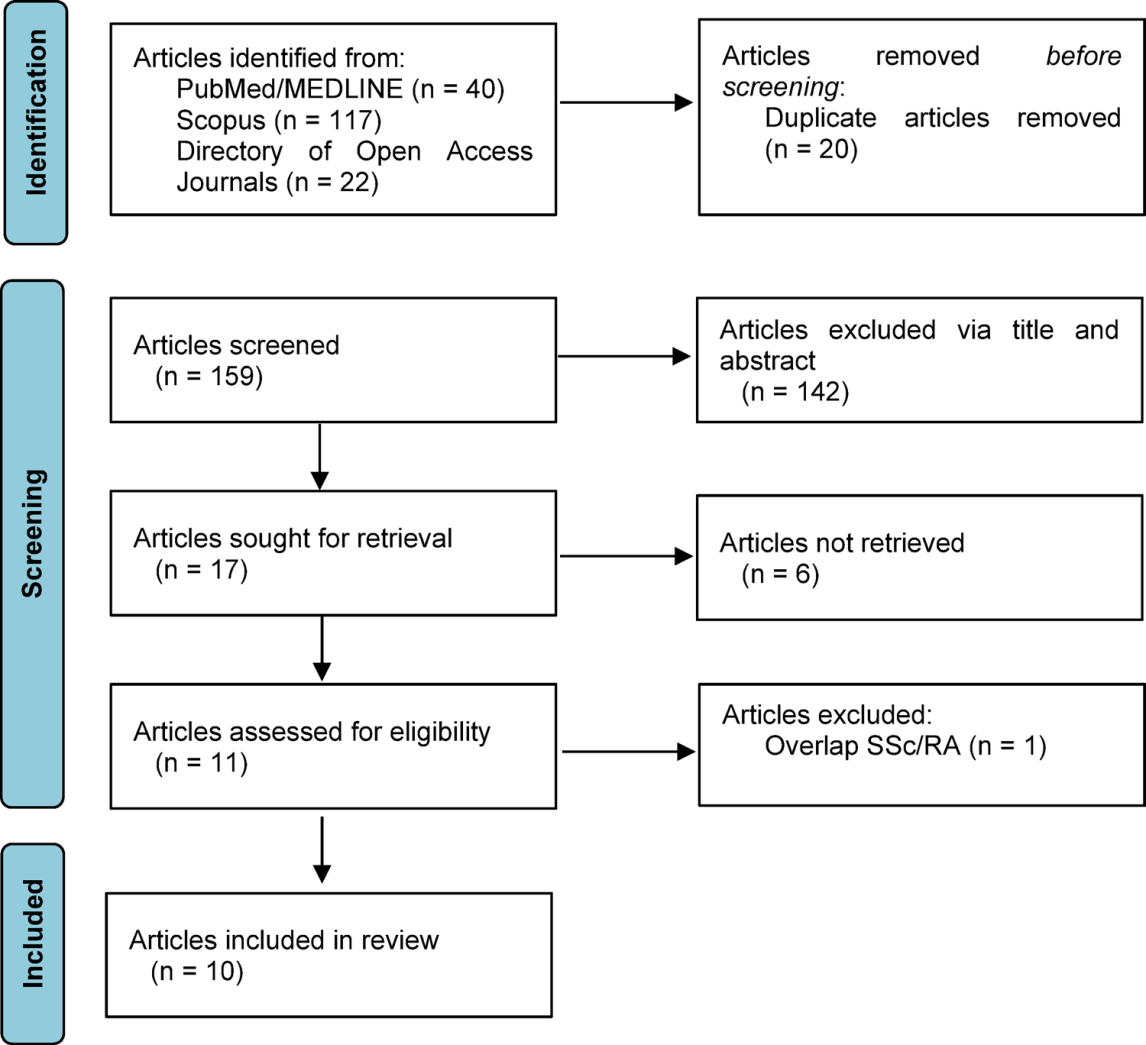
PRISMA 2020 flow diagram of study selection. Flow diagram showing the number of records identified, screened, excluded, and included in the qualitative and quantitative synthesis according to PRISMA 2020 guidelines

This case-based review was conducted in accordance with the CABARET (CAse-BAsed REview sTandards) recommendations of the EQUATOR Network [8]. We conducted a literature search in PubMed/MEDLINE, Scopus, and the Directory of Open Access Journals without restrictions on publication date (up to July 20, 2025). The keywords used for the search were: “systemic sclerosis”, “scleroderma”, and “amyloidosis.” All peer-reviewed case series and case reports describing the concomitant presence of SSc and amyloidosis were included. Articles not published in English were excluded. Two authors (A.C. and C.P.) independently performed the literature review, screened titles and abstracts, and assessed the full texts of potentially eligible articles. Duplicates were removed. Disagreements between the authors were resolved through discussion. A flow diagram summarizing the selection process is provided (Fig. 4).

## Discussion

SSc is a rare disease characterized by autoimmunity, fibrosis of the skin and internal organs, and vasculopathy. Affected organs include the skin, lungs, kidneys, and heart. While some manifestations of SSc are insidious and progress slowly, others may present acutely and progress rapidly [9].

Amyloidosis includes a heterogeneous group of disorders characterized by abnormal deposition of insoluble protein aggregates in extracellular spaces, leading to organ dysfunction. Amyloid is composed of protein subunits that adopt a beta-sheet configuration, rendering them insoluble. These aggregates stain with Congo red and display apple-green birefringence under polarized light [10]. Subtypes of amyloidosis are classified by the type of amyloid fibrils, such as AL (light chain), AH (heavy chain), and AA (serum amyloid A) [11].

We identified 13 reported cases of SSc associated with amyloidosis (Table 1). Most patients were female, with a mean age around the fifth to sixth decade of life and predominantly presented with the limited cutaneous subtype of SSc. Autoantibody profiles were heterogeneous but often included anticentromere antibodies (ACA).

The majority (9/13) involved primary amyloidosis with exclusively cutaneous deposition, while only one case described tendinous involvement. Notably, cases describing major organ involvement such as the heart or kidneys were limited to secondary amyloidosis, which is rarely reported in SSc compared to other chronic inflammatory diseases (e.g., rheumatoid arthritis or systemic lupus erythematosus). To our knowledge, our case is the first describing a patient fulfilling the 2013 EULAR/ACR classification criteria for systemic sclerosis who developed primary systemic amyloidosis.

**Table 1:**
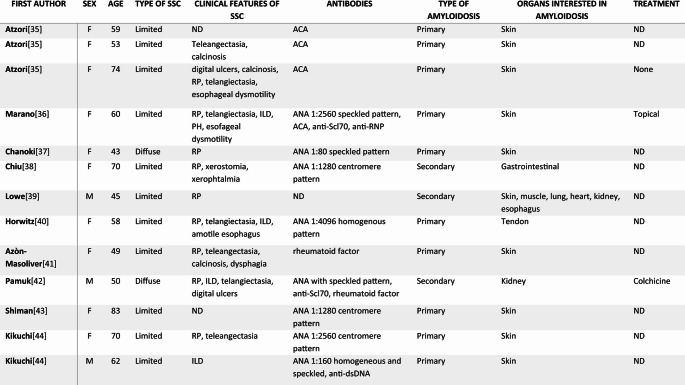
Published cases of systemic sclerosis (SSc) associated with amyloidosis

### SSc and AL amyloidosis: comparative clinical manifestations

Few cases have been reported where amyloidosis (usually AA) was misdiagnosed as SSc or occurred in conjunction with the autoimmune disease in the literature, highlighting the complexity in distinguishing between these conditions [12].

Bély and Apáthy [13] performed a histopathological analysis of 12 deceased SSc patients depicting AL deposits in 1 patient (8.0%). This was a 67 years-old female patient with a 1-year disease history of SSc associated with B-cell lymphoma and complicated by systemic AL-amyloidosis. They found that the prevalence and extent of light-chain deposits were higher in contrast to amyloid A, probably due to a greater affinity of AL for tissues than amyloid A protein. Infiltration of the vessel walls - regarding the amount of light-chain and amyloid A deposits in arterioles and arteries in contrast to the veins - showed an inverse tendency in SSc patients with AL or AA. This may be related to sluggish blood flow or stasis (backward congestion and accumulation of circulating precursors) in both diseases.

However, there are no cases describing the simultaneous onset of SSc and AL amyloidosis and their management. Diagnosis can be challenging since both diseases may affect the same organs while possibly exhibiting distinct disease-specific alterations.

Skin involvement in SSc typically begins in the distal extremities and can extend proximally, involving the face and trunk, while AL amyloidosis presents with more variable cutaneous manifestations such as petechiae, purpura, nodules, and rarely scleroderma-like lesions. Cases of scleredema preceding or coexisting with AL amyloidosis and multiple myeloma have been reported, typically without SSc-specific autoantibodies and lacking clinical features such as RP or ILD, features that, by contrast, are present in our patient [14, 15]. In addition to amyloidosis, a scleroderma-like cutaneous pattern can be caused by drugs, exposure to toxic agents, nephrogenic systemic fibrosis, thyroid disorders such as hyperthyroidism or hypothyroidism, and paraneoplastic syndromes, all of which were excluded in our patient [16]. 

Pulmonary involvement in SSc usually manifests as ILD with a nonspecific interstitial pneumonia (NSIP) pattern, especially in anti-Scl70-positive patients. AL amyloidosis can affect the lungs through diffuse tracheobronchial deposits, nodular parenchymal amyloidosis (amyloidomas), or diffuse alveolar-septal involvement, which may mimic SSc-related ILD and complicate differential diagnosis [10, 17].

Cardiac manifestations also differ: SSc may present with myocardial inflammation, perfusion defects, and fibrosis (more frequent in anti-Scl70-positive patients), while AL amyloidosis typically causes restrictive cardiomyopathy due to interstitial amyloid deposition, with ventricular thickening and preserved ejection fraction. Advanced imaging techniques, such as assessment of myocardial deformation and late gadolinium enhancement, help distinguish the two conditions and have prognostic value in AL amyloidosis [10, 18–20].

### Therapeutic approaches for SSc and AL amyloidosis

Current treatment for AL amyloidosis focuses on reducing the production of amyloidogenic immunoglobulin light chains by targeting plasma cell clones, aiming to decrease amyloid precursors and halt further deposition. This approach allows gradual tissue regression of amyloid deposits, improving organ function and survival. Therapeutic options include ASCT and, for ineligible patients, Dara-CyBorD regimen [21].

Treatment for SSc varies according to the organ affected. Recently, ACR/EULAR updated recommendations have been proposed [22]. Effective options include methotrexate, rituximab (RTX), MMF, and tocilizumab for skin involvement, and CYC, RTX, or MMF for ILD. Cardiac manifestations may be managed with corticosteroids and CYC in cases of myocarditis or life-threatening arrhythmias [18, 22, 23].

In the last two decades, B cells have been recognized as central to autoimmunity. Anti-CD20 therapy with RTX benefits many autoimmune diseases but often fails to eliminate long-lived plasma cells that continue producing pathogenic autoantibodies. Daratumumab, an anti-CD38 monoclonal antibody shows promise in refractory autoimmune diseases such as systemic lupus erythematosus, Sjögren’s syndrome, and ANCA-associated vasculitis. By directly depleting long-lived, autoantibody-secreting plasma cells, daratumumab may achieve deeper and more durable disease control than B cell–targeted therapies alone [24–27].

Beyond its role in plasma cells, CD38 is overexpressed in patients with SSc, a disease marked by widespread fibrosis. CD38 promotes the breakdown of nicotinamide adenine dinucleotide (NAD+), leading to lower intracellular NAD + levels and reduced activity of sirtuins, which normally act to restrain fibrotic processes. Experimental studies show that inhibiting CD38, genetically or pharmacologically, or restoring NAD + levels through precursor supplementation, significantly reduces fibrosis in multiple organs by restoring sirtuin activity and dampening pro-fibrotic signalling. These findings highlight CD38 as an active mediator of fibrosis in SSc, suggesting it could represent a novel therapeutic target to counteract tissue fibrosis [28–31].

Among established treatments for SSc and amyloidosis, CYC has long been used in neoplastic and autoimmune disorders for its cytotoxic and immunosuppressive effects and remains indicated in SSc with severe organ involvement.

Additionally, bortezomib has shown antifibrotic effects by blocking TGF-β1-mediated target gene expression, thereby promoting normal tissue repair and preventing skin and lung fibrosis after injury. This drug was tested in a phase II trial on patients with SSc. The results have not yet been published, but are available at https://clinicaltrials.gov/study/NCT02370693?tab=results, and suggest a potential benefit when used in combination with MMF compared to placebo [32].

While the importance of ASCT for the treatment of AL amyloidosis is well established, emerging evidence supports its use in patients with SSc presenting with early and diffuse cutaneous involvement, accompanied by organ involvement, which is associated with a 50% 5-year survival rate [22, 33, 34]. However, as abovementioned, this strategy was not suitable for our patient.

MMF was chosen as maintenance therapy to help control disease activity, given the good response of amyloidosis to Dara-CyBorD regimen and the lack of robust data on daratumumab use in SSc. It could be of interest to explore the potential of daratumumab in monotherapy for maintenance of SSc, besides this drug seems to be more suitable for induction of response and long term regimes appear to be limited by potential side effects including hypogammaglobulinemia [23].

## Limitations

This case-based review describes the clinical response of a single patient with SSc and AL amyloidosis. As such, the single-case design limits the generalizability of our findings to broader patient populations. Furthermore, we lacked histological or immunophenotypic confirmation of plasma cell infiltration or CD38 expression in the affected tissues, which would have strengthened the link between treatment and response in SSc. Thus, while the clinical improvement observed is of interest, these results should be viewed as preliminary and interpreted with caution, as they do not establish a definitive therapeutic effect.

## Conclusions

To the best of our knowledge, this report represents the first description of a case of SSc associated with systemic AL amyloidosis, highlighting the diagnostic complexity and the importance of targeted therapy.

Treatment for amyloidosis demonstrated improvement in SSc manifestations, suggesting potential shared therapeutic benefits.

A multidisciplinary approach was crucial for the diagnosis and management of these rare conditions. This case underscores the need for further research to understand the interactions between these diseases and optimize diagnostic and therapeutic strategies.

## Declarations

### Congress abstract

No related congress abstract has been published.
